# Comparative analysis of the complete plastomes of nine *Pimpinella* species (Apiaceae) from China

**DOI:** 10.7717/peerj.14773

**Published:** 2023-02-28

**Authors:** Zhixin Wang, Limin Cao, Jianhui Liu, Xingjin He

**Affiliations:** 1 Hunan Key Laboratory for Conservation and Utilization of Biological Resources in the Nanyue Mountainous Region, College of Life Sciences and Environment, Hengyang Normal University, Hengyang, Hunan, China; 2 Key Laboratory of Bio-Resources and Eco-Environment of Ministry of Education, College of Life Sciences, Sichuan University, Chengdu, Sichuan, China

**Keywords:** Apiaceae, Chloroplast genome, *Pimpinella*, Phylogenetic relationship

## Abstract

*Pimpinella* L. is one of the large genera in the Apiaceae family. In a previous study, the molecular phylogenies of *Pimpinella* were explored using nuclear ribosomal DNA internal transcribed spacers (ITS) and several chloroplast DNA segments. There have been few studies conducted on chloroplast genomes in *Pimpinella*, which has limited systematic understanding of this genus. We assembled the complete chloroplast genomes of nine *Pimpinella* species from China using data generated from next generation sequencing (NGS). The chloroplast (cp) DNA used were standard double-stranded molecules, ranging from 146,432 base pairs (bp) (*P. valleculosa*) to 165,666 bp (*P. purpurea*) in length. The circular DNA contained a large single-copy (LSC) region, small single-copy (SSC) region, and pair of inverted repeats (IRs). The cp DNA of the nine species contained 82–93 protein-coding genes, 36–37 transfer RNA (tRNA) genes, and eight ribosomal RNA (rRNA) genes, respectively. Four species (*P. smithii*, *P. valleculosa*, *P. rhomboidea*, and *P. purpurea*) exhibited striking distinctions in genome size, gene number, IR boundary, and sequence identity. We confirmed the non-monophyly of the *Pimpinella* species on the basis of the nine newly identified plastomes. The distant relationship between the above-mentioned four *Pimpinella* species and Pimpinelleae was indicated with high support values. Our study provides a foundation for future in-depth phylogenetic and taxonomic studies of genus *Pimpinella*.

## Introduction

The genus *Pimpinella* L. (Apiaceae, Apioideae) is comprised of approximately 150 species that are mainly distributed throughout Europe, Africa, and Asia, with 39 species and two varieties found in China (Pu, 1985). This total was recently revised to include 44 species from the flora of China (Pu & Watson, 2005). Its members are characterized by ordinary ovate and laterally compressed mericarps without wings or spines on the ribs. The boundaries between the species within this genus are obscure. Moreover, the generic borders between *Pimpinella* and its related genera (such as *Carum*, *Apium*, *Aegopodium*, and *Spuriopimpinella*) are frequently blurred. The published molecular phylogenies of *Pimpinella* were inferred using the nuclear ribosomal internal transcribed spacer (ITS) region and a few plastid markers (*rps16* intron, *rpl16* intron, *rps16* exon, *trnL* intron, and *trnL-F* spacer) (Magee et al., 2010; Wang et al., 2014; Fereidounfar, Ghahremaninejad & Khajehpiri, 2016; Fernández Prieto et al., 2018; Mousavi et al., 2022). The genus *Pimpinella* is not monophyletic and its members have been included among the Selineae, Echinophoreae, Pyramidoptereae, and *Acronema* clades and the East Asian clade (Wang et al., 2014; Mousavi et al., 2022).

A study of 26 *Pimpinella* species from China was conducted using data from the ITS and two cpDNA intron sequences (Wang et al., 2014), many parallel branches and lower support values were identified. There is a need to find more suitable polymorphic regions. In recent years, many comparative analyses of chloroplast genomes have enriched the molecular phylogenetic study of Apiaceae (Kang et al., 2019; Guo et al., 2020; Li et al., 2020; Ren et al., 2020; Liu et al., 2022). Few chloroplast genomes of the *Pimpinella* species have been reported (Tan & Yu, 2018; Wang, Cao & Liu, 2020) and the current comparative analysis of *Pimpinella* from China at the whole chloroplast genome level is still lacking. We sought to further investigate the plastome features of the *Pimpinella* species and enrich the phylogeny of the complicated *Pimpinella* genus. The nine complete plastid genome sequences of the newly sequenced *Pimpinella* will lay the foundation for understanding the relationships among these common Chinese *Pimpinella* members.

## Materials & Methods

### Sample collection, DNA extraction, and genome sequencing

In this study, we investigated nine *Pimpinella* species distributed throughout China (Table 1). Voucher specimens were preserved in the herbarium of Hengyang Normal University (HYNU). Fresh leaves collected in the field were dried and stored in silica. Total genomic DNA were then extracted using a modified cetyltrimethylammonium bromide protocol (Doyle, 1987), which was conducted by Novogene (Tianjin, China). DNA library prep and sequencing were completed at Novogene (Tianjin, China) on the Illumina Novaseq platform using the PE150 sequencing strategy.

### Plastome sequence assembly and annotation

Utilizing NOVOPlasty 3.7 (Dierckxsens, Mardulyn & Smits, 2017) with the ribulose-1,5-bisphosphate carboxylase/oxygenase (*rbc* L) gene from *Pimpinella diversifolia* DC. (GenBank accession number MT561033) as the seed file, the raw reads of genome-related chloroplasts were assembled. After aligning with congeneric species in pairs, the plastome sequences were annotated using Geneious Prime 2019.2.3 (Kearse et al., 2012), supplemented with necessary manual adjustment. Nine plastome sequences accompanied with their annotations were submitted to NCBI (ON321877 –ON321885).

### Chloroplast genome comparative analyses

In each of the nine plastome sequences, two inverted repeat (IR) regions were determined using the ‘Repeat Finder’ plugin in Geneious Prime (Kearse et al., 2012). Utilizing ‘Extract Annotations,’ all protein-coding genes (CDS) were extracted in Geneious Prime (Kearse et al., 2012) and subsequently concatenated into one sequence for each accession. We removed genes with lengths shorter than 300 bp, as well as repeated gene sequences. Using CodonW (https://sourceforge.net/projects/codonw/), the relative synonymous codon usage (RSCU) values of each species were determined. These RSCU values were presented on a heatmap created using TBtools v1.086 (Chen et al., 2020). With the help of an online software tool named PREP (Mower, 2009), RNA editing sites were forecasted.

REPuter (Kurtz et al., 2001) was utilized to search for the short-dispersed repeats (SDRs) of nine plastome sequences. All four options (forward, reverse, complement, and palindromic repeats) were chosen for ‘Match Direction’. Moreover, the Hamming distance value was set to 3 and minimal repeat size was set to 30. Using the MIcroSAtellite identification tool (MISA) (https://webblast.ipk-gatersleben.de/misa/) (Beier et al., 2017), simple sequence repeats (SSRs) were identified. Before that, 10, five, four, three, three, and three were selected as the minimum number of SSRs for mono-, di-, tri-, tetra-, penta-, and hexanucleotides, respectively.

**Table 1:** Plant accessions from sequences that were investigated in this study.

Accession no./taxon name	Sample site	Location	Voucher no.
*Pimpinella candolleana* Wight et Arn.	Kunming, Yunnan	25°8′38″N, 102°44′34″E	w190804
*P. diversifolia* DC.	Hangzhou, Zhejiang	30°20′43″N, 119°26′43″E	w2101
*P. purpurea* (Franch.) de Boiss.	Lijiang, Yunnan	27°2′42″N, 100°11′47″E	w19080103
*P. rhomboidea* Diels	Wushan, Chongqing	31°17′11″N, 110°4′26″E	w20082201
*P. rubescens* (Franch.) Wolff ex Hand.-Mazz.	Lijiang, Yunnan	27°3′17″N, 100°11′46″E	w19080102
*P. scaberula (Franch.)* Wolff	Deqin, Yunnan	28°1′9″N, 98°54′1″E	w20081301
*P. smithii* Wolff	Wushan, Chongqing	31°17′11″N, 110°4′26″E	w20082202
*P. thellungiana* H. Wolff	Lvliang, Shanxi	37°14′21″N, 111°12′42″E	w190825
*P. valleculosa* K.T.Fu	Wenxian, Gansu	32°49′52″N, 105°23′39″E	H92903

We used the online tool IRSCOPE (https://irscope.shinyapps.io/irapp/) to display the structure of the nine plastomes, and adjusted manually if necessary. Utilizing the MAFFT (Katoh & Standley, 2013) plugin (alignment algorithm of FFT-NS-2 selected) within Geneious Prime (Kearse et al., 2012), nine *Pimpinella* plastomes were aligned. After that, the genetic distance of these nine *Pimpinella* plastomes was estimated using MEGA-X (https://www.megasoftware.net/) and the Kimura 2-parameter model (Kumar et al., 2018).

### Phylogenetic analysis

In order to investigate the relationship between the nine *Pimpinella* species and allied taxa within Apioideae, we conducted phylogenetic analysis on the chloroplast genome sequences. Before that, the complete plastomes of the 13 other species within Apioideae of related genera were downloaded from the NCBI: *Angelica sylvestris*, *Bupleurum boissieuanum*, *Bupleurum falcatum*, *Bupleurum latissimum*, *Cnidium officinale*, *Crithmum maritimum*, *Hansenia forbesii*, *Hansenia weberbaueriana*, *Haplosphaera phaea*, *Peucedanum praeruptorum*, *Pleurospermum camtshaticum*, *Pterygopleurum neurophyllum,* and *Tongoloa silaifolia* (see GenBank accession numbers in the Supplemental File). The dataset was subsequently aligned using MAFFT (with algorithm of FFT-NS-2) in Geneious Prime (Kearse et al., 2012). Maximum likelihood (ML) analysis was performed using raxmlGUI (Silvestro & Michalak, 2012) with rapid bootstrap (1,000 replicates) and the GTRGAMMA model.

## Results

### The total characteristics of cp DNA of nine *Pimpinella* species

The complete cp genome of the nine *Pimpinella* species was a double-stranded molecule that ranged from 146,432 bp in *P. valleculosa* to 165,666 bp in *P. purpurea*. The cp genome had a conserved structure, identical to the majority of angiosperm plants, that contained a pair of IR regions (IRa and IRb), one LSC region, and one SSC region (Table 2). The plastid genomes of *P. candolleana*, *P. diversifolia*, *P. rubescens*, *P. scaberula*, and *P. thellungiana* were comprised of 130 genes: 84 protein-coding genes, 37 tRNA genes, and eight rRNA genes. Among these, 96 genes were unique, and 17 genes were duplicated in the IR region, including six protein-coding genes (*ndhB, rpl23, rps7, rps12, ycf1,* and *ycf2*), seven tRNA genes (*trnA-UGC, trnI-CAU, trnI-GAU, trnL-CAA, trnN-GUU, trnR-ACG*, and *trnV-GAC*), and four rRNA genes (*rrn4.5, rrn5, rrn16*, and *rrn23*).

Moreover, the plastid genomes of *P. smithii* and *P. valleculosa* contained 127 genes: 82 protein-coding genes, 36 tRNA genes, and eight rRNA genes. Among the 127 genes, 99 were unique and 14 genes were duplicated in the IR region, including four protein-coding genes (*ndhB, rps7, rps12,* and *ycf1*), six tRNA genes (*trnA-UGC, trnI-GAU, trnL-CAA, trnN-GUU, trnR-ACG*, and *trnV-GAC*) and four rRNA genes (*rrn4.5, rrn5, rrn16*, and *rrn23*).

The plastid genomes of *P. rhomboidea* consisted of 131 genes, including 85 protein-coding genes, 37 tRNA genes, and eight rRNA genes. Among these, 95 genes were unique and 18 genes were duplicated in the IR region, including seven protein-coding genes (*ndhB, rpl2, rpl23, rps7, rps12, ycf1,* and *ycf2*), seven tRNA genes (*trnA-UGC, trnI-CAU, trnI-GAU, trnL-CAA, trnN-GUU, trnR-ACG*, and *trnV-GAC*) and four rRNA genes (*rrn4.5, rrn5, rrn16*, and *rrn23*).

The plastid genomes of *P. purpurea* consisted of 139 genes, including 93 protein-coding genes, 37 tRNA genes and eight rRNA genes. Among these, 87 genes were unique and 26 genes were duplicated in the IR region, including 15 protein-coding genes (*infA*, *ndhB, rpl2, rpl14, rpl16, rpl22, rpl23, rpl36, rps3, rps7, rps8, rps12, rps19, ycf1,* and *ycf2*), seven tRNA genes (*trnA-UGC, trnI-CAU, trnI-GAU, trnL-CAA, trnN-GUU, trnR-ACG*, and *trnV-GAC*), and four rRNA genes (*rrn4.5, rrn5, rrn16*, and *rrn23*).

**Table 2:** The features of chloroplast genomes of nine *Pimpinella* species.

Species	Size (bp)	LSC (bp)	SSC (bp)	IR (bp)	Number of protein-coding genes	Number of tRNA genes	Number of rRNA genes	GC content (%)
*P. candolleana*	153,199	86,109	17,137	24,913	84	37	8	37.7
*P. diversifolia*	153,135	86,219	17,114	24,901	84	37	8	37.7
*P. purpurea*	165,666	86,110	17,458	31,049	93	37	8	37.9
*P. rhomboidea*	153,298	85,662	16,754	25,441	85	37	8	37.7
*P. rubescens*	152,236	85,209	17,113	24,957	84	37	8	37.6
*P. scaberula*	152,499	85,645	17,120	24,867	84	37	8	37.6
*P. smithii*	147,183	93,487	17,486	18,105	82	36	8	37.5
*P. thellungiana*	152,558	85,271	17,063	25,112	84	37	8	37.8
*P. valleculosa*	146,432	93,553	17,473	17,703	82	36	8	37.5

### Codon usage and RNA editing site prediction

**Figure 1:**
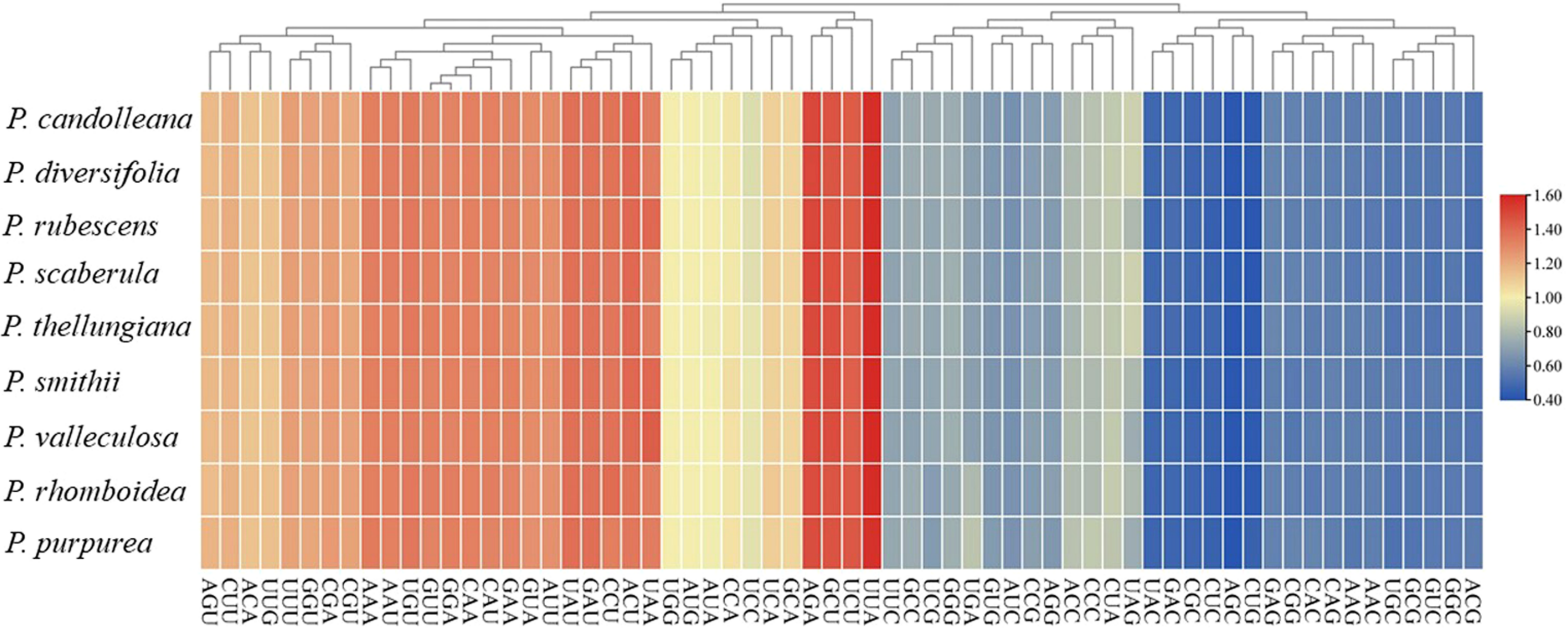
The RSCU values of codons in the nine *Pimpinella* plastomes.

A total of 53 protein-coding genes were extracted from the nine *Pimpinella* plastomes. The total sequence sizes of these genes for codon analysis ranged from 63,423–63,516 bp. These protein sequences encoded 21,141–21,172 codons (Table 3). The GC content of the 1st, 2nd, and 3rd position of codons were 46.1, 38.3, and 29.8%, respectively. The RSCU values of the 30 codons were more than 1 (Table S1), and almost all of these ended with A/U. Approximately half of the codons were used more frequently, as shown in Fig. 1. These plastomes had an obvious bias in the use of codons towards the third position containing A/U. The amino acid leucine (Leu) had the largest number of codons (2,215–2,244), while cysteine (Cys) had the lowest number of codons (218–223).

**Table 3:** The indexes of the codon usage bias in the nine *Pimpinella* species.

Index\Taxon	*P. candolleana*	*P. diversifolia*	*P. rubescens*	*P. scaberula*	*P. thellungiana*	*P. smithii*	*P. valleculosa*	*P. rhomboidea*	*P. purpurea*
Length (bp)	63,516	63,513	63,447	63,516	63,516	63,432	63,483	63,435	63,423
Codon No.	21,172	21,171	21,149	21,172	21,172	21,144	21,161	21,145	21,141
Amino acid No.	21,119	21,118	21,096	21,119	21,119	21,091	21,108	21,092	21,088
SC No.	20,259	20,258	20,235	20,261	20,262	20,230	20,252	20,223	20,222
ENC	49.80	49.84	49.73	49.73	49.76	49.74	49.68	49.72	49.97
CAI	0.165	0.165	0.165	0.165	0.166	0.167	0.166	0.166	0.167
CBI	−0.103	−0.103	−0.101	−0.102	−0.102	−0.098	−0.099	−0.101	−0.099
FOP	0.352	0.352	0.353	0.353	0.353	0.355	0.355	0.354	0.355
GC content (%)	0.381	0.382	0.381	0.381	0.382	0.380	0.380	0.381	0.383

In an analysis of the RNA editing sites, a total of 516 RNA editing sites were identified. The smallest number of editing sites was 56 for *P. purpurea* and the greatest was 58 in *P. rhomboidea*, *P. smithii*, *P. thellungiana*, and *P. valleculosa* (Table S2). In all of the nine *Pimpinella* plastomes, the *ndhB* gene contained the largest RNA editing sites (10 or 11). All of the RNA editing sites belonged to the same conversion type (cytosine to uracil (C-U)). Among these, the majority (41–45) were located in the 2nd codon position, and the remainder (13–17) were in the 1st position.

### Repeats analysis

**Figure 2:**
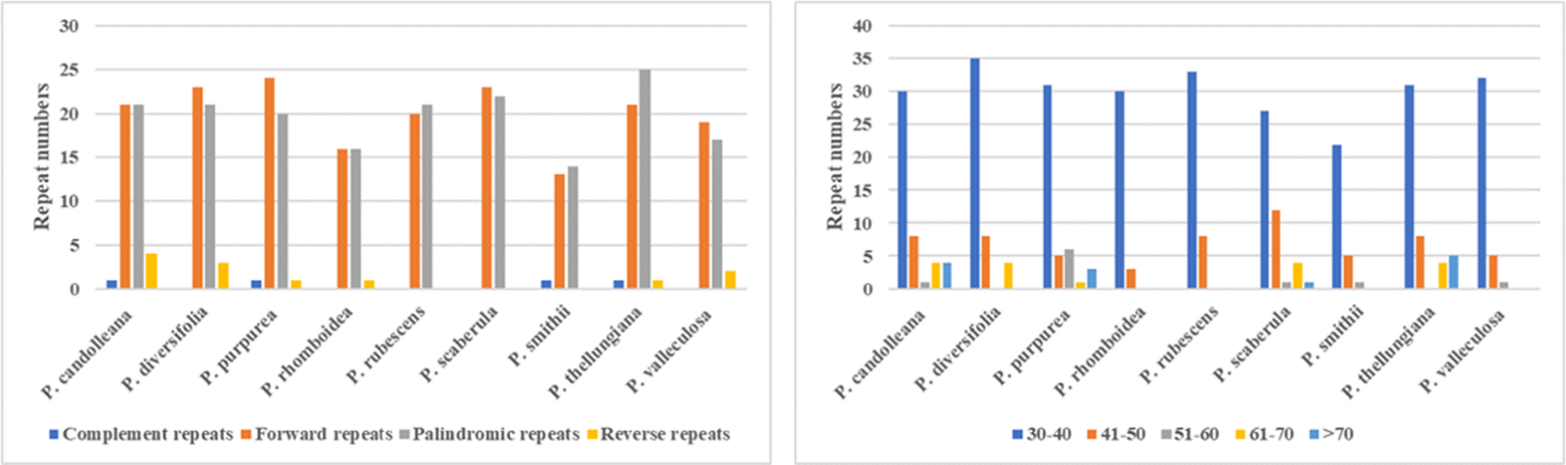
The comparison of SDRs analysis in the nine *Pimpinella* plastomes.

**Figure 3:**
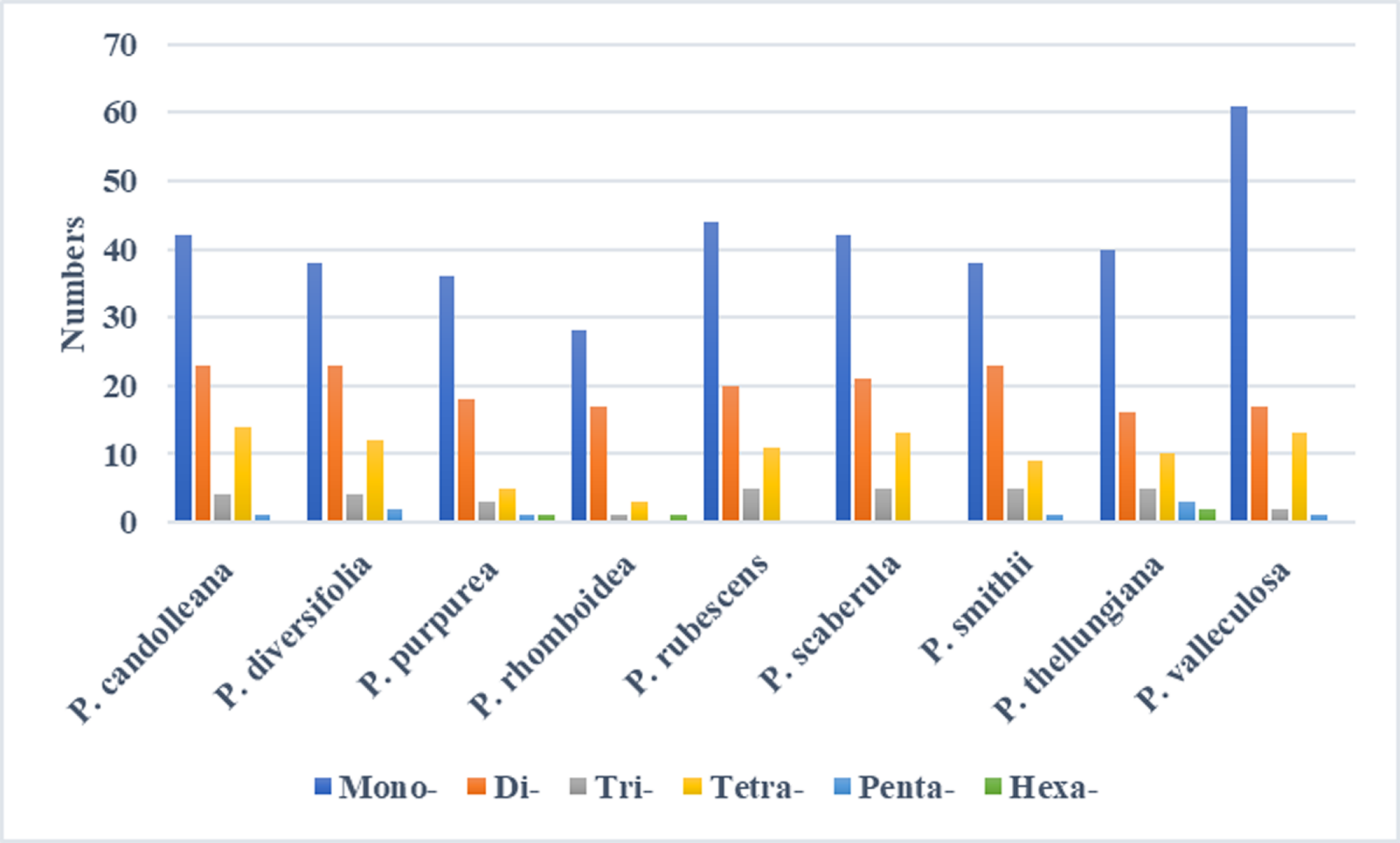
The identification of SSRs in the nine *Pimpinella* plastomes.

In the SDRs analysis, four types of repeats were determined: forward, reverse, complement, and palindromic repeats. Forward and palindromic repeats occurred in a large proportion in the nine *Pimpinella* plastomes. Moreover, the repeats with lengths of 30-40 bp accounted for the greatest proportion (Fig. 2). In the SSRs analysis, 50-94 SSRs were identified in the nine *Pimpinella* plastomes. The number of single nucleotide repeats was the largest (Fig. 3), with the majority being A/T repeats.

### IR boundary comparison

**Figure 4:**
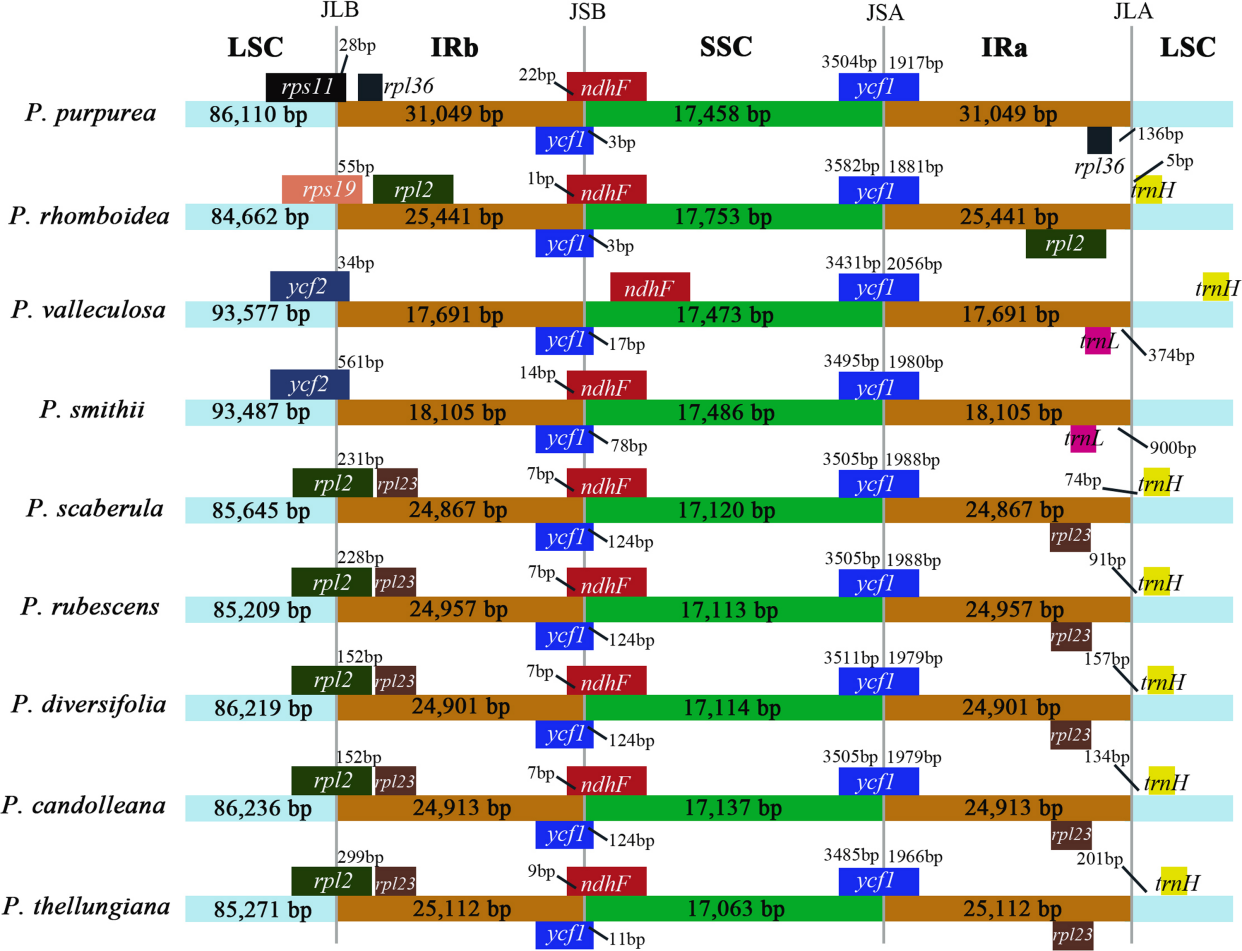
The IR boundary comparison in the nine *Pimpinella* plastomes.

The comparison of IR/SC borders among the nine plastomes are shown in Fig. 4. The gene arrangements and contents in the IR/SC borders of the plastomes of these five species (*P. candolleana*, *P. diversifolia*, *P. rubescens*, *P. scaberula,* and *P. thellungiana*) coincided. The LSC/IRb border of *P. smithii* and *P. valleculosa,* located in the *ycf2* gene, and the *trnL* gene were 900 bp and 374 bp away from the IRa/LSC border, respectively. The LSC/IRb border of *P. rhomboidea,* located in the *rps19* gene, and the *trnH* gene was five bp away from the IRa/LSC border. The LSC/IRb border of *P. purpurea,* located in the *rps11* gene, and the *rpl36* gene had a distance of 136 bp from the IRa/LSC border.

### Genetic distance analysis

Genetic distance analysis results are shown in Table 4. The highest value of pairwise genetic distance was 0.0365 between *P. candolleana* and *P. purpurea*, while the lowest was 0.0007 between *P. candolleana* and *P. diversifolia*.

**Table 4:** The pairwise distance of chloroplast genomes from nine *Pimpinella* species.

	*P. rubescens*	*P. scaberula*	*P. diversifolia*	*P. candolleana*	*P. thellungiana*	*P. valleculosa*	*P. smithii*	*P. rhomboidea*
*P. scaberula*	0.0019							
*P. diversifolia*	0.0048	0.0046						
*P. candolleana*	0.0048	0.0047	0.0007					
*P. thellungiana*	0.0116	0.0116	0.0115	0.0115				
*P. valleculosa*	0.0211	0.0211	0.0208	0.0209	0.0201			
*P. smithii*	0.0206	0.0205	0.0203	0.0204	0.0197	0.0070		
*P. rhomboidea*	0.0350	0.0352	0.0353	0.0353	0.0347	0.0311	0.0308	
*P. purpurea*	0.0362	0.0364	0.0365	0.0365	0.0358	0.0319	0.0318	0.012

### Phylogeny

**Figure 5:**
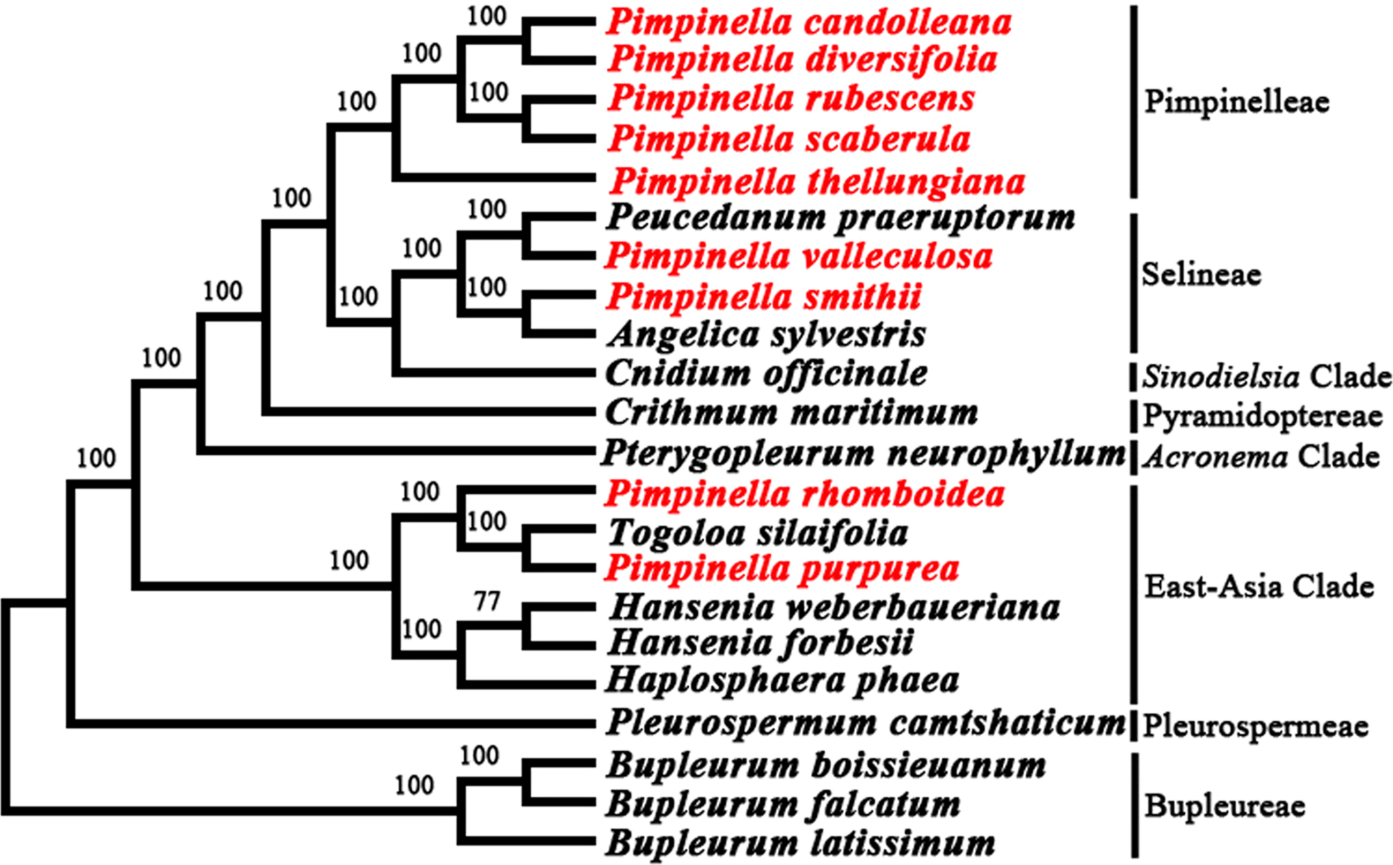
Phylogenetic analysis from the plastomes of nine *Pimpinella* species and allied taxa.

The phylogenetic relationship between nine *Pimpinella* species and related taxa in the Apioideae was well resolved with high support values (Fig. 5) based on the whole plastome sequence data. It was indicated that five *Pimpinella* species fell in the tribe Pimpinelleae. *P. smithii* and *P. valleculosa* were located in the tribe Selineae. *P. rhomboidea* and *P. purpurea* were clustered in the East-Asian clade, which coincided with the results obtained from ITS and cpDNA sequences (*rps16* and *rpl16* introns) (Wang et al., 2014).

## Discussion

Apiaceae is a family that is famous for its unique fruit characteristics. *Pimpinella* is a relatively large member of the Apiaceae family and understanding the phylogeny and taxonomy of this genus is important. In this study, the *Pimpinella* plastomes were determined to be all quadripartite in the genome structure. We determined it had several distinct characteristics. First, the overall sizes of chloroplast genomes varied from 146,432 bp (*P. valleculosa*) to 165,666 bp (*P. purpurea*). Second, the numbers of the unique genes encoded by plastomes ranged from 87 (*P. purpurea*) to 99 (*P. smithii* and *P. valleculosa*). Among the closely-related species, the plastomes rarely fluctuated in size and gene content. For example, the plastome sizes of nine *Chamaesium* species reported (Guo et al., 2020) ranged from 152,703 bp to 155,712 bp, and contained 133 genes uniformly (including 95 unique genes). As another example, the chloroplast genome lengths of five *Bupleurum* species ranged from 155,621 bp to 156,108 bp, with similar genome structure (Li et al., 2020). In most cases, the variations in the lengths of the plastomes resulted from the contraction and expansion of the IR region (Downie & Jansen, 2015). IR contraction of ∼1.5 kb in *Pimpinella* relative to tobacco (Plunkett & Downie, 2000) was suggested based on chloroplast-DNA restriction site analysis. Herein, *Pimpinella candolleana*, *P. diversifolia*, *P. rubescens*, *P. scaberula*, and *P. thellungiana* with IR sizes of 24,867–25,112 bp possessed ∼0.2–0.4 kb IR contraction relative to tobacco (*Nicotiana tabacum*) (GenBank accession No. Z00044). Most notably, the large contractions of the IR regions in *P. smithii* (∼7.2 kb) and *P. valleculosa* (∼7.6 kb) made their plastome sizes significantly smaller than the newly identified plastomes. Subsequently, in *P. smithii* and *P. valleculosa*, *rps19*, *rpl2*, *rpl23,* and *ycf2* had only one copy. The huge expansion (∼5.7 kb) of the IR regions in *P. purpurea* with a length of 31,049 bp made it the largest of the nine newly-obtained *Pimpinella* plastomes. At the LSC/IRb border, its IR expanded ∼6.1 kb compared with *P. diversifolia*, and resulted in many genes (*infA*, *rpl2, rpl14, rpl16, rpl22, rpl36, rps3, rps8,* and *rps19*) contained within the IR region. This kind of large IR expansion (>1,000 bp) was not usual, and also happened to *Crithmum maritimum* (Apiaceae) (Downie & Jansen, 2015). The expansion may originate from sequence recombination (Downie & Jansen, 2015), although this is waiting to be confirmed by subsequent studies.

The preference for codons ending with A/T in the *Pimpinella* plastomes was confirmed and also observed in other genera in Apiaceae (Guo et al., 2020; Li et al., 2020; Ren et al., 2020; Liu et al., 2022). The conversion type (C-U) of the RNA editing sites found here was similar to that of many other vascular plants (Wakasugi, Tsudzuki & Sugiura, 2001). Short repeats with 30-40 bp occupied most of these editing sites, which was consistent with the *Ligusticum* species in Apiaceae (Ren et al., 2020). A large number of SSRs in the newly sequenced *Pimpinella* chloroplast genomes were discovered to be mononucleotides (A/T), similar to many other angiosperms.

Our analyses support the results of previous studies based on ITS and cp DNA marker (*rps16* intron and *rpl16* intron) sequences (Wang et al., 2014). These studies found that the Chinese *Pimpinella* congeners did not cluster in one group, but instead mainly gathered in the *Pimpinella* core group, accompanied by *P. smithii* and *P. valleculosa* clustered within Selineae. *P. purpurea* and *P. rhomboidea* diverged earlier and clustered within the East Asian clade. The results clearly show *Pimpinella* as one non-monophyletic group. Meanwhile, our plastome data included a large amount of phylogenetically informative characteristics with greater support for the present topology compared to previous ones (Wang et al., 2014). They also provide a basis for future taxonomic studies of Chinese *Pimpinella*. In China, *Pimpinella thellungiana* is the most closely-related to the type species (*P. saxifraga* L.) (Wang et al., 2014). Four species clustered with *P. thellungiana* should be moved into the *Pimpinella sensu stricto* clade. Moreover, perhaps *P. smithii* should be transferred into the genus *Angelica* and *P. valleculosa* into *Peucedanum*. As for *P. purpurea* and *P. rhomboidea*, the present results were aligned with the treatments (Gui L, 2022, unpublished) to combine these two species into *Tongoloa*. The greatly divergent plastomes of *P. purpurea* and *P. rhomboidea*, compared to the other *Pimpinella* plastomes examined, may be related to potential chloroplast capture events. ‘Capture’ cases (Rieseberg & Soltis, 1991) have been reported in many different taxa. Chloroplast capture may bring incongruent plastid and nuclear DNA phylogeny reconstruction (Liu et al., 2020). It is necessary to be cautious when completing phylogenetic reconstruction using chloroplast DNA data (Rieseberg, Choi & Ham, 1991). Although chloroplast capture is also present in Apiaceae (Yi, Jin & Wen, 2015; Wen et al., 2021), the improper classification of *P. purpurea and P. rhomboidea* within genus *Pimpinella* may be a more accurate explanation, considering their congruent phylogenetic relationships between nuclear and chloroplast phylogenies (Wang et al., 2014). These taxonomic changes depend on a broader sampling for molecular analyses and morphological studies.

Previous studies (Guo et al., 2020; Li et al., 2020; Ren et al., 2020; Liu et al., 2022) have shown promising results in using the complete chloroplast genome sequences to infer phylogenies of genera in Apiaceae. Our study indicated that the plastomes proved to be good markers when used to reconstruct the phylogeny with a larger sampling. In addition, the *Pimpinella* and allied taxa may have a more complicated phylogeny, and future in-depth studies based on the chloroplast genomes are necessary.

## Conclusion

This study is the first attempt to comprehensively examine plastome features and infer phylogeny using plastome data for the *Pimpinella* genus. The circular DNA of nine newly obtained plastomes contained a LSC region, SSC region, and pair of IRs. The plastomes ranged from 146,432 bp (*P. valleculosa*) to 165,666 bp (*P. purpurea*) in length. The plastid genomes of *P. candolleana*, *P. diversifolia*, *P. rubescens*, *P. scaberula*, and *P. thellungiana* were comprised of 130 genes, including 84 protein-coding genes, 37 tRNA genes, and eight rRNA genes. However, *P. smithii* and *P. valleculosa* contained only 127 genes. Moreover, the plastome of *P. rhomboidea* consisted of 131 genes. Most intriguingly, the plastid genome of *P. purpurea* consisted of 139 genes, including 93 protein-coding genes, accompanied by 15 protein-coding genes(*infA*, *ndhB, rpl2, rpl14, rpl16, rpl22, rpl23, rpl36, rps3, rps7, rps8, rps12, rps19, ycf1,* and *ycf2*), and seven tRNA genes (*trnA-UGC, trnI-CAU, trnI-GAU, trnL-CAA, trnN-GUU, trnR-ACG*, and *trnV-GAC*) duplicated. Phylogenetic analysis revealed that *P. candolleana*, *P. diversifolia*, *P. rubescens*, *P. scaberula*, and *P. thellungiana* were clustered in Pimpinelleae; *P. smithii* and *P. valleculosa* were clustered with Selineae members; and *P. rhomboidea* and *P. purpurea* in the East-Asian clade were relatively distant from their congeners. This study provides new evidence that chloroplast genomes might be useful when reconstructing the phylogeny of *Pimpinella* with a larger species sampling, which will be helpful for solving taxonomy problems in the genus.

##  Supplemental Information

10.7717/peerj.14773/supp-1 Table S1 The number and RSCU values of 64 codons in the nine *Pimpinella* species

10.7717/peerj.14773/supp-2 Table S2 The RNA editing sites analysis

10.7717/peerj.14773/supp-3 Table S3 List of GenBank accession numbers of all taxa used in the phylogenetic analysis

10.7717/peerj.14773/supp-4 Supplemental Information 1 Sequence data

## Additional Information and Declarations

### DNA Deposition

The following information was supplied regarding the deposition of DNA sequences: The plastomes sequences are available in the Supplemental File and at GenBank: ON321877–ON321885.

### Data Availability

The following information was supplied regarding data availability: The raw reads of all the nine plastomes are available at NCBI Short Read Archive (SRA): PRJNA836345.
